# Assessment of Asymptomatic Severe Aortic Regurgitation by Doppler-Derived Echo Indices: Comparison with Magnetic Resonance Quantification

**DOI:** 10.3390/jcm11010152

**Published:** 2021-12-28

**Authors:** Zuzana Hlubocká, Radka Kočková, Hana Línková, Alena Pravečková, Jaroslav Hlubocký, Gabriela Dostálová, Martin Bláha, Martin Pěnička, Aleš Linhart

**Affiliations:** 1 Department of Cardiovascular Medicine, General University Hospital, 12808 Prague, Czech Republic; gabriela.dostalova@vfn.cz (G.D.); ales.linhart@vfn.cz (A.L.); 2 Department of Cardiology, Institute for Clinical and Experimental Medicine, 14021 Prague, Czech Republic; radka.kockova@homolka.cz (R.K.); alena.praveckova@ikem.cz (A.P.); martin.blaha@ikem.cz (M.B.); 3 Department of Cardiology, Royal Vinohrady University Hospital, 10034 Prague, Czech Republic; hana.linkova@fnkv.cz; 4 Department of Cardiovascular Surgery, General University Hospital, 12808 Prague, Czech Republic; jaroslav.hlubocky@vfn.cz; 5 Onze-Lieve-Vrouwziekenhuis Aalst Clinic, Cardiovascular Centre Aalst, 9300 Aalst, Belgium; martin.penicka@olvz-aalst.be

**Keywords:** aortic regurgitation, echocardiography, magnetic resonance, quantification, vena contracta

## Abstract

Reliable quantification of aortic regurgitation (AR) severity is essential for clinical management. We aimed to compare quantitative and indirect echo-Doppler indices to quantitative cardiac magnetic resonance (CMR) parameters in asymptomatic chronic severe AR. Methods and Results: We evaluated 104 consecutive patients using echocardiography and CMR. A comprehensive 2D, 3D, and Doppler echocardiography was performed. The CMR was used to quantify regurgitation fraction (RF) and volume (RV) using the phase-contrast velocity mapping technique. Concordant grading of AR severity with both techniques was observed in 77 (74%) patients. Correlation between RV and RF as assessed by echocardiography and CMR was relatively good (r_s_ = 0.50 for RV, r_s_ = 0.40 for RF, *p* < 0.0001). The best correlation between indirect echo-Doppler and CMR parameters was found for diastolic flow reversal (DFR) velocity in descending aorta (r_s_ = 0.62 for RV, r_s_ = 0.50 for RF, *p* < 0.0001) and 3D vena contracta area (VCA) (r_s_ = 0.48 for RV, r_s_ = 0.38 for RF, *p* < 0.0001). Using receiver operating characteristic analysis, the largest area under curve (AUC) to predict severe AR by CMR RV was observed for DFR velocity (AUC = 0.79). DFR velocity of 19.5 cm/s provided 78% sensitivity and 80% specificity. The AUC for 3D VCA to predict severe AR by CMR RV was 0.73, with optimal cut-off of 26 mm^2^ (sensitivity 80% and specificity 66%). Conclusions: Out of the indirect echo-Doppler indices of AR severity, DFR velocity in descending aorta and 3D vena contracta area showed the best correlation with CMR-derived RV and RF in patients with chronic severe AR.

## 1. Introduction

Management of asymptomatic patients with chronic severe aortic regurgitation (AR) and preserved left ventricular (LV) ejection fraction (EF) is often challenging. Reliable quantification of AR severity is of crucial importance for guiding clinical management and improving clinical outcome [1,2]. Although such patients typically remain asymptomatic for a long time, they should be promptly referred to surgery once either symptoms or LV dysfunction develop [3,4,5].

Transthoracic echocardiography is recognized as the primary imaging modality for initial assessment and longitudinal evaluation of patients with chronic AR. Grading of AR severity is achieved by an integrative approach using several qualitative, semiquantitative, and quantitative parameters [5,6]. Three-dimensional echocardiography provides more precise assessment of LV volumes and function. Furthermore, 3D color Doppler echocardiography measures the 3D vena contracta area (VCA) and proximal isovelocity surface area (PISA) and may increase accuracy of AR quantification [7,8].

However, echocardiographic quantification can be challenging because of poor acoustic windows, inconclusive Doppler data, eccentric jets, and geometric assumptions. Current ESC guidelines indicate that cardiac magnetic resonance (CMR) should be used to quantify AR when echocardiography is inconclusive [5]. CMR is considered the gold standard technique for evaluation of ventricular volumes and function [9]. It can directly measure regurgitant volume (RV) and regurgitant fraction (RF) using the technique of phase-contrast velocity mapping [10,11]. This method has been correlated with long-term clinical outcome in severe AR [12]. However, CMR-specific cut-off values for RV and RF, which identify significant AR, are not clearly established. Quantitative thresholds for RV and RF are mostly derived from echocardiography.

Because there are only limited data on direct comparison between TTE and CMR for quantification of AR [13,14], we aimed to compare quantitative and indirect echo-Doppler indices to quantitative CMR-derived parameters of AR severity (RV and RF) in asymptomatic patients with severe chronic AR.

## 2. Materials and Methods

### 2.1. Study Population

The study population consisted of consecutive patients diagnosed with moderate-to-severe (3+ grade) and severe (4+ grade) chronic AR according to echocardiography assessment. Patients were enrolled between March 2016 and January 2019 in three tertiary referral cardiac centers. All patients were asymptomatic, did not meet the indication criteria for surgical treatment according to current clinical guidelines, and were in sinus rhythm. Exclusion criteria included any other associated moderate or severe valvular disease, history of coronary artery disease, atrial fibrillation, creatinine clearance <30 mL/min/1.73 m^2^, poor echocardiography image quality, and contraindication to CMR.

Clinical history, presence of symptoms, physical examination, electrocardiography, and blood samples were assessed. Cardiac magnetic resonance and TTE exams were performed <7 days apart (median 2 (0, 10) days).

The study was conducted in accordance with the Declaration of Helsinki, and the study protocol was approved by the ethics committee of the General University Hospital, Prague (project 85/16 Grant VES 2017 AZV VFN). All subjects gave their written informed consent prior to enrollment.

### 2.2. Echocardiography

A comprehensive 2D and 3D TTE was performed using the Vivid 95 and Vivid 9 imaging systems (GE Healthcare, Chicago, IL, USA) equipped with two-dimensional (2D) and three-dimensional active matrix 4D volume phased array probe. Several 3D echo loops in each view were recorded using ECG-gated full-volume acquisition over four to six cardiac cycles during end-expiratory apnea. Blood pressure and heart rate were recorded during each examination. All acquired data were digitally stored, anonymized, and analyzed off-line using commercially available software (EchoPAC Sofware Version 203, GE Vingmed Ultrasound AS, Horten, Norway) in a central laboratory of the Institute of Clinical and Experimental Medicine, Prague, which holds the European Association of Cardiovascular Imaging (EACVI) Laboratory accreditation and individual certification. Evaluation was performed by a single experienced cardiologist (R.K.) blinded to CMR data.

Left ventricular dimensions at end-diastole and end-systole were measured using 2D method in parasternal long axis (PLAX) view. LV volumes and EF were calculated by the biplane disc summation method (modified Simpson’s rule) [15]. The area of the LV outflow tract (LVOT) and mitral valve were calculated from 2D and 3D images.

**Figure 1:**
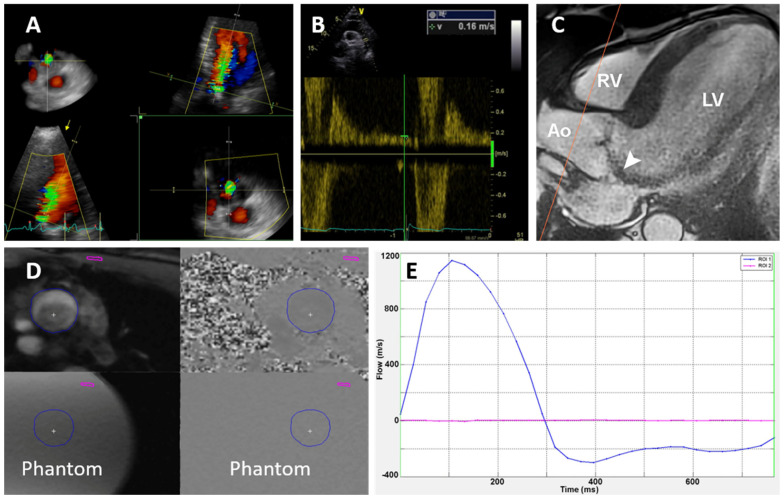
Imaging methods. (**A**) 3D vena contracta area as assessed by 3D echocardiography. (**B**) Diastolic flow reversal velocity in descending aorta measured by pulsed-wave Doppler. (**C**) Cardiac magnetic resonance; through-plane flow sequence slice placed in ascending aorta (red line). (**D**) Through-plane flow sequence. The blue circle is a manually drawn region of interest where the blood flow, regurgitant volume, and fraction were calculated. Phantom: stationary phantom used for flow measurement correction. (**E**) Flow-time curve based on (**D**). The blue line shows the flow in the aorta, and the red line shows the flow in the stationary phantom.

Severity of AR was graded using a recommended multiparametric approach based on integration of several parameters as proposed in recent recommendations [5,16]. These measures included valve morphology, jet width in LVOT and jet width to LVOT width ratio (percentage), vena contracta width, jet density and deceleration rate, and presence and velocity of diastolic flow reversal (DFR) in proximal descending aorta measured by pulsed-wave Doppler method (Figure 1B). We intended to primarily use the PISA method to quantify RV and effective regurgitant orifice area (EROA). Unfortunately, PISA was not feasible in our study population in about 50% of patients, most frequently due to eccentricity of jets and valve calcifications in bicuspid valves. Therefore, all RV and RF measurements presented in the manuscript were performed using the Doppler volumetric method, which uses differences between the mitral and aortic stroke volume [6,16].

Other assessed measurements included aortic dimensions, velocity time integral and gradient over the aortic and mitral valve, right ventricular size and function, and tricuspid regurgitation gradient. Three-dimensional TTE was used to assess LV volumes and function, 3D LVOT, and mitral anulus area. Moreover, 3D echo-derived vena contracta area (3D VCA) was measured using 3D color Doppler echocardiography in zoomed PLAX view as described previously [17,18] (Figure 1A).

### 2.3. Cardiac Magnetic Resonance

The CMR studies were performed in one center (Institute of Clinical and Experimental Medicine, Prague, Czech Republic) using a 1.5 Tesla scanner (Magnetom Avanto fit, Siemens Medical Systems, Erlangen, Germany). Protocol scans included pilots, T2 weighted dark blood, cine with 2-, 4-, 3-chamber and short-axis images, contrast late gadolinium enhancement, and through-plane phase-contrast velocity mapping at the aortic root level. CMR image analysis was done by a single CMR specialist blinded to echocardiography data using the research version of image software Segment (Medviso AB 2018, Lund, Sweden).

Left ventricle ejection fraction and volumes were assessed in short axis using cine imaging with correction for valve position. The through-plane phase-contrast velocity mapping technique was used to evaluate aortic forward and regurgitation flow during respiration apnea with retrospective ECG gating. Several image slices were made in the ascending aorta at end-diastole starting from 0.5 cm above the aortic annulus to 0.5 cm above the sinotubular junction (thickness of slice 6 mm, spacing 0). The slices were aligned perpendicularly to the blood flow in two orthogonal planes. We chose the lowest velocity encoding without aliasing for the calculations. Using flow stationary phantom and post-processing correction, the background velocity offset errors were corrected. To decrease underestimation of RV and RF, the closest slice to the aortic annulus without the turbulent flow was used for analysis as recommended [7,13,19]. Aortic RV and forward stroke volume (SV) were calculated by integrating the flow curve. The regurgitant fraction (RF) was calculated as RV/SV × 100 (%) (Figure 1C–E) [13,14].

### 2.4. Statistical Analysis

Statistical analyses were performed using the statistical package SPSS version 20 software (SPSS Inc., Chicago, IL, USA) and GraphPad Prism version 6.0 (GraphPad Software, San Diego, CA, USA).

Data are expressed as mean ± standard deviation (SD) for continuous variables except for BNP and as counts or percentages for categorical variables. BNP was summarized using median and interquartile range. Correlations between continuous echocardiographic and CMR variables were assessed by Spearman’s correlation coefficient (r_s_). Bland–Altman plots were used to compare differences in RV and RF between echocardiography and CMR-derived measurements.

Receiver operating characteristic (ROC) analyses with corresponding areas under the curve (AUC) were used to determine the Doppler echocardiographic parameters to predict severe aortic regurgitation by CMR RV and RF. The optimal cut-off values for sensitivity and specificity were calculated according to the Youden’s index and according to clinical relevance. For all comparisons, a *p* value <0.05 was considered statistically significant.

## 3. Results

### 3.1. Baseline Clinical and Imaging Characteristics

A total of 104 from 107 eligible patients were enrolled in the study. Three patients (2.8%) failed to complete CMR study (claustrophobia or spine deformity). Two-dimensional TTE study with satisfactory image quality was successfully completed in all patients. Baseline clinical and demographic characteristics of the study population are shown in Table 1. The echocardiographic and CMR imaging data are summarized in Table 2. Mean patient age was 44 ± 13 years, and 89 (86%) patients were male. Of note, 83 patients (81%) had bicuspid, unicuspid, or quadricuspid aortic valves. The most prevalent comorbidity was hypertension (48%) with corresponding medication. Blood pressure was well controlled in all patients. All patients were asymptomatic with preserved LV systolic function and sinus rhythm.

A total of 56 (53.8%) individuals had moderate-to-severe (3+) AR, while the remaining 48 (46.2%) showed severe (4+) AR according to integrative echocardiographic approach. Concordant grading of AR severity with both techniques was observed in 77 (74%) patients. An integrative TTE approach showed a trend to underestimate AR severity. A total of 17 patients with 3+ AR as per TTE assessment had severe AR by CMR quantification.

### 3.2. Comparison of Quantitative Echo and CMR Parameters

Correlation coefficients between quantitative parameters measured by echo and CMR are listed in Table 3. Quantitative parameters of AR severity (RV and RF) as assessed by the 2D TTE volumetric method and CMR correlated relatively well with each other (r_s_ = 0.50, *p* < 0.0001 for RV and r_s_ = 0.40, *p* < 0.0001 for RF). Quantification of RV and RF using 3D echocardiography did not significantly improve the correlation (r_s_ = 0.49, *p* < 0.0001 for RV and r_s_ = 0.52, *p* < 0.0001 for RF).

**Figure 2:**
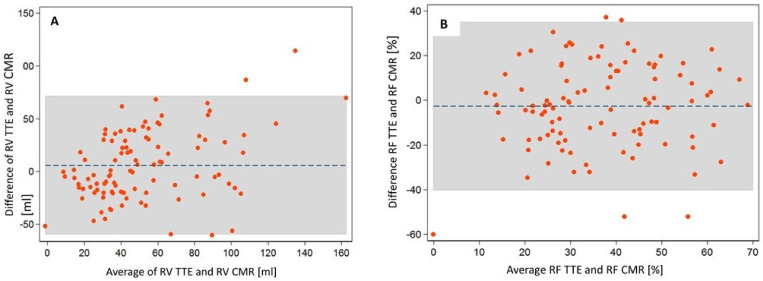
Bland–Altman plots comparing (**A**) the regurgitation fraction (RF) and (**B**) the regurgitation volume (RV) obtained by echocardiography (TTE) and CMR (cardiac magnetic resonance). The average of the two methods is plotted against the difference in RF (**A**) or RV (**B**) as assessed by TTE and CMR.

The Bland–Altman plots showed slightly larger RV and lower RF as measured by the 2D-TTE volumetric method compared to the CMR method. Mean 2D-TTE-derived RV was 5.7 mL larger and RF was 2.7% lower than the CMR-derived parameters; 95% limits of agreement were relatively wide (Figure 2).

### 3.3. Comparison of Indirect Echo Indices to Quantitative CMR Parameters

Out of indirect echocardiographic Doppler indices of AR severity, DFR velocity in proximal descending aorta and 3D VCA showed good correlation with CMR-derived RV (r_s_ = 0.62 and 0.48, respectively, *p* < 0.001,) and RF (r_s_ = 0.50 and 0.38, respectively, *p* < 0.0001).

On the contrary, 2D-TTE vena contracta width showed nonsignificant correlations with CMR parameters (r_s_ = 0.18 and *p* = 0.07 for RV, r_s_ = 0.05 and *p* = 0.62 for RF).

### 3.4. Echo Parameters Predicting Severe AR by CMR Quantification

The diagnostic value of semiquantitative echo-derived parameters to identify severe AR as assessed by CMR quantification (RV >50 mL and RF >30%) was analyzed using receiver operating characteristic analysis.

**Figure 3:**
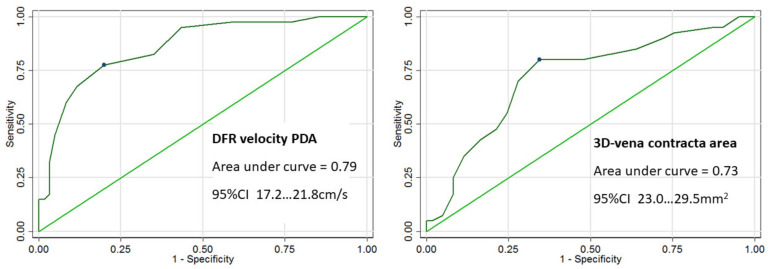
Receiver operating characteristics curves with corresponding areas under the curve and confidence intervals (CI) of the diastolic flow reversal velocity in proximal descending aorta (DFR velocity PDA) and 3D vena contracta area (3D VCA) to predict severe AR (regurgitation volume ≥50 mL) using cardiac magnetic resonance.

Out of the echo-derived indices of AR severity, the largest area under the curve (AUC) to predict severe AR by CMR RV was observed for DFR velocity in the descending aorta (AUC = 0.79) and 3D VCA (AUC = 0.73). Velocity of DFR in proximal descending aorta of 19.5 cm/s provided the best sum of sensitivity and specificity (78% sensitivity and 80% specificity) with 95% confidence interval (17.2–21.8 cm/s) (Figure 3). The optimal cut-off value of 3D VCA to predict severe AR by CMR RV was ≥26 mm^2^, with sensitivity of 80%, specificity of 66%, and confidence interval (23.0–29.5 mm^2^).

The velocity of DFR in the descending area and 3D VCA to predict severe AR by CMR-derived RF fraction showed smaller AUC (0.72 and 0.64, respectively).

## 4. Discussion

In a cohort of asymptomatic patients with moderate-to-severe and severe aortic regurgitation, we found DFR velocity in descending aorta and 3D VCA to be most closely correlated with the quantitative measures of AR severity (RF and RV) determined by CMR phase-contrast flow measurements.

In addition, we found relatively good concordance between the CMR-based AR severity quantification and echocardiography grading of AR severity using an integrative approach. A direct comparison of RV and RF measured by CMR and volumetric echocardiography method revealed moderately good correlation.

### 4.1. Quantitative Echocardiographic Parameters

Due to known limitations of echocardiography (quality of imaging, number of achievable visualization planes in 2D, and operator-related variability in interpretation), the quantification of AR severity by TTE is based on integration of a set of quantitative, semiquantitative, and qualitative parameters. Recent guidelines strongly support the use of quantitative methods (mainly the PISA method) and provide thresholds for AR severity grading [6,16]. The prognostic value of these thresholds has been validated and shown to outperform semiquantitative methods [20]. However, in patients with eccentric regurgitant jets or valve calcifications, good-quality continuous and color Doppler recordings are frequently difficult to achieve. This situation may be particularly frequent in individuals with congenitally abnormal aortic valve. Moreover, the often-asymmetric character of regurgitant orifice may decrease the accuracy of the PISA method.

Because we were unable to perform PISA volumetric assessments in a significant number of subjects, we used a quantification of RV and RF by the Doppler volumetric method. We demonstrated relatively good correlation between this approach and values obtained by CMR. Compared to the CMR method, the mean RV by TTE was slightly larger and RF slightly lower. Several reports have attempted to correlate the severity of AR measured by CMR and TTE regurgitation volume and fraction. Overall, correlation in these studies was moderately good [13,21,22]. There are many potential reasons for the observed differences. Several studies reported good reproducibility for transaortic stroke volume measurements. In contrast, there was a wide variability for measurement of mitral annulus diameter and transmitral velocity–time integral assessment [21,23]. Precise and reproducible measurement of mitral annulus diameter on TTE is difficult. Moreover, measuring the diameter and velocity at the same anatomic site is challenging, resulting in overestimation or underestimation of flow.

A potential source of error might also be related to CMR assessments. During the phase-contrast CMR measurements, some blood ejected into the ascending aorta that has not yet crossed the image slice and flows back into the LV is not measured as part of the regurgitant volume [24]. Moreover, the CMR quantification relies on a laminar flow in the ascending aorta, and the presence of eccentric and turbulent jets in bicuspid valves can lead to variation in flow measurements. Phase-contrast CMR also has relatively low temporal resolution, so the peak systolic velocity and forward flow may be underestimated, particularly in concomitant aortic stenosis [10,25].

These differences between CMR and echocardiographic quantification of RV and RF could explain the higher RV and lower RF as assessed by echocardiography compared to the CMR phase-contrast method. Other studies have also reported higher AR regurgitation volume as measured by echocardiography compared to CMR-derived RV [10,21,22].

Due to PISA limitations and the complexity of volumetric measurements, the evaluation of AR severity in routine clinical practice often remains based on semiquantitative methods, including vena contracta, DRF in descending aorta, pressure half-time, and others.

### 4.2. Indirect Echocardiographic Parameters

Diastolic backward flow in proximal descending aorta mirrors the regurgitant volume. The current recommendations consider pulsed-wave DRF velocity in descending aorta to be a strong additional parameter to assess AR severity [16]. Our study showed a good correlation of DFR velocity in descending aorta with quantitative CMR parameters. According to ROC analysis, DFR velocity of 19.5 cm/s shows good sensitivity and specificity to predict severe AR by CMR (CMR RV >50 mL).

Several studies report good correlation of DFR velocity in descending aorta with other echocardiographic parameters of AR severity, including quantitative assessment by the PISA method [6,26].

Furthermore, Bolen reported that holodiastolic flow reversal in the descending aorta evaluated using through-plane phase contrast CMR correlated well with echocardiographic diagnosis of severe AR, although DFR was observed less frequently on CMR than with TTE. The authors suggested that this difference might be due to the aortic level assessed (mid-descending aorta with CMR vs. proximal descending aorta with TTE) [27].

An abnormally stiff or dilated aorta was hypothesized to dampen DFR in some patients. However, Bolen et al. showed that aortic stiffness and aortic geometry were similar in patients with and without DRF, indicating that DRF is affected more by AR severity than by the size and elastic properties of the aorta [27]. It remains debatable whether blood pressure levels may significantly influence the DFR. The blood pressure of our patients was well controlled. Previous studies have not shown that vasodilator therapy can decrease the regurgitant volume, improve EF, or delay the need for surgery [28]. Thus, we believe that treatment with ACE inhibitors and other antihypertensive drugs did not influence the AR severity assessment.

In a recent study by Kammerlander et al., the presence of holodiastolic flow reversal in the descending aorta as assessed by CMR was an important parameter that predicted clinical outcome in patients with severe AR [28].

Although the bicuspid aortic valve is the most frequent cause of primary aortic insufficiency, its prevalence in our study population was very high. To enable good comparison between echocardiographic and CMR parameters of AR severity, exclusion criteria of our study comprised any other more than mild valvular disease, coronary artery disease, atrial fibrillation, or decompensated hypertension. Thus, we probably excluded many patients with other etiology of AR, namely degenerative mixed aortic valve disease, secondary aortic regurgitation, or rheumatic valve diseases. Therefore our cohort of patients was not representative of the general population. The study population included mainly asymptomatic younger patients with early stage of severe aortic regurgitation disease, which more frequently present with bicuspid aortic valves.

As discussed above, one of the difficulties in bicuspid aortic valves is the asymmetric character of the regurgitant jet and irregular shape of the regurgitant orifice. This might also explain why 2D vena contracta width did not correlate with CMR parameters of AR severity. Three-dimensional data can be rotated perpendicular to the jet direction to avoid the limitation introduced by jet eccentricity, and planimetry overcomes the elliptical shape of the jet cross-section. Three-dimensional VCA is a 3D-derived parameter of the vena contracta size that does not rely on geometric assumptions. Three-dimensional VCA has been shown to be highly accurate, reproducible, and superior to the PISA method in several native valve regurgitation studies [7,29,30]. Our study demonstrates that 3D VCA has a good correlation with CMR-derived parameters of AR severity (RV and RF). We identified the optimal cut-off value of 3D VCA at >26 mm^2^. This value was associated with CMR-measured RV >50 mL with 80% sensitivity and 66% specificity. Previous studies have reported higher cut-off values of 3D VCA to predict severe AR based on the integrative TTE approach. Chin et al. reported that the cut-off value of 3D VCA was >50 mm^2^, while Sato et al. found 3D VCA >32 mm^2^ as an optimal cut-off value for severe AR. Three-dimensional VCA was also shown to predict future cardiac surgery in patient with AR with optimal cut-off of ≥30 mm^2^ [31]. These thresholds need to be confirmed in further prospective studies.

### 4.3. Clinical Implications

Accurate assessment of the aortic regurgitation (AR) severity is important for clinical decision-making. Echocardiographic quantification of AR is comprehensive, always considering several semiquantitative and quantitative parameters. Severe AR is typically characterized by dilatation of left ventricle, vena contracta ≥6 mm, end-diastolic flow reversal velocity in descending aorta ≥20 cm/s, RV ≥60 mL, and EROA ≥30 mm^2^ as assessed by the PISA method. Although the importance of quantitative parameters measured by the PISA method is emphasized in the guidelines, they are not always feasible or easy to perform in daily clinical practice. Confirmation of AR severity using other imaging methods is therefore reassuring. Asymptomatic patients with severe AR are indicated for surgery if LV end-systolic diameter is >50 mm (indexed diameter >25 mm/m^2^) or LV ejection fraction is ≤50%. Lower thresholds may be considered in selected patients with low surgical risk.

Cardiac magnetic resonance (CMR) should be used to evaluate AR severity when echocardiographic parameters are equivocal or when there is a discrepancy between AR severity as assessed by echocardiography and clinical symptoms. Moreover, CMR as the gold standard method to assess LV systolic function is appropriate when accurate evaluation of LV ejection fraction and volumes is needed for optimal timing of surgery.

## 5. Study Limitations

Our study is limited by its cross-sectional character. We did not assess the prognostic significance of echo and CMR-derived parameters in terms of need for valve surgery and other outcomes in this study. Longitudinal follow-up of the cohort is ongoing. The study cohort is not representative of the general population of patients with aortic regurgitation as discussed above.

## 6. Conclusions

Echocardiography remains the primary method to evaluate AR severity and guide clinical care in most patients. Cardiac magnetic resonance provides highly reproducible and accurate quantification of AR using the phase-contrast velocity method. However, the lower availability, higher costs, and presence of contraindications make CMR unlikely to replace TTE.

Therefore, it is important to find echo parameters that best correlate with CMR quantitative parameters of AR severity. In our study, PW end-diastolic velocity in descending aorta over 19.5 cm/s and vena contracta area over 26 mm^2^ as measured by 3D echocardiography showed the best power to predict severe AR by CMR quantification. Further investigation is required to correlate echo and CMR assessment of AR and set CMR thresholds for AR quantification.

## Figures and Tables

**Table 1:** Baseline clinical characteristics of study population (*n* = 104).

Variable	Value (%)
Age, years	44 ± 13
Male gender, N (%)	89 (86)
Hypertension, N (%)	50 (48)
Diabetes mellitus, N (%)	6 (6)
Hyperlipidemia, N (%)	29 (28)
Smoker, N (%)	14 (13)
Coronary artery disease, N (%)	4 (4)
Previous cardiac surgery, N (%)	4 (4)
Stroke, N (%)	1 (1)
NYHA Class I, N (%)	104 (100)
Height, cm	180 ± 9
Weight, kg	85 ± 14
Systolic blood pressure, mmHg	136 ± 16
Diastolic blood pressure, mmHg	70 ± 12
Heart rate, beats per min	64 ± 10
B natriuretic peptide, ng/L	27 (42)
Hemoglobin, g/L	153 ± 13

Values are means ± standard deviations, median (interquartile range), or numbers (percentage). ACEI/ARB: angiotensin-converting enzyme inhibitor/angiotensin receptor blocker, NYHA: New York Heart Association.

**Table 2:** Baseline imaging characteristics of study population (*n* = 104).

Variable	Value
Aortic valve morphology	
Trileaflet, N (%)	14 (13.6)
Bicuspid, N (%)	79 (76.7)
Unicuspid/quadricuspid, N (%)	4 (4)
Unknown, N (%)	6 (6)
Aortic regurgitation assessment	
Integrative approach	
Moderate-to-severe AR, N (%)	56 (54)
Severe AR, N (%)	48 (46)
2D echo vena contracta width, mm	6.5 ± 1.5
Diastolic flow reversal velocity, cm/s	19.4 ± 4.3
2D echo regurgitant volume, mL	52 ± 48
2D echo regurgitant fraction, %	36 ± 18
3D echo vena contracta area, mm^2^	29 ± 13
CMR regurgitation volume, mL	50 ± 28
CMR regurgitation fraction, %	38 ± 17
Left ventricle assessment	
2D echo end-diastolic diameter, mm	58 ± 6
2D echo end-systolic diameter, mm	37 ± 5
2D echo end-systolic diameter index, mm/m^2^	18 ± 3
2D echo end-diastolic volume, mL	158 ± 68.0
2D echo end-systolic volume, mL	56 ± 32
2D echo ejection fraction, %	64 ± 6
3D echo end-diastolic volume, mL	177 ± 51
3D echo end-diastolic volume index, mL/m^2^	86 ± 23
3D echo end-systolic volume, mL	69 ± 24
3D echo end-systolic volume index, mL/m^2^	33 ± 11
3D echo ejection fraction, %	62 ± 5
CMR end-diastolic volume, mL	234 ± 81
CMR end-diastolic volume index, mL/m^2^	118 ± 30
CMR end-systolic volume, mL	88 ± 51
CMR end-systolic volume index, mL/m^2^	43 ± 23
CMR ejection fraction, %	61 ± 6

Values are means ± standard deviations or numbers (percentage). 2D: two-dimensional, 3D: three-dimensional, echo: echocardiography, CMR: cardiac magnetic resonance.

**Table 3:** Correlation between parameters of AR severity using echocardiography (TTE) and cardiac magnetic resonance (CMR).

Variable	r_s_	*p* Value
Regurgitant volume by CMR		
vena contracta width	0.18	0.07
3D VCA	0.48	<0.001
DFR velocity in descending aorta	0.62	<0.001
RV by TTE volumetric method	0.50	<0.001
RF by TTE volumetric method	0.44	<0.001
Regurgitant fraction by CMR		
vena contracta width	0.05	0.62
3D VCA	0.38	<0.001
DFR velocity in descending aorta	0.50	<0.001
RV by TTE volumetric method	0.43	<0.001
RF by TTE volumetric method	0.40	<0.001
LV EDV by CMR and by TTE	0.78	<0.001
LV ESV by CMR and by TTE	0.73	<0.001
LV ejection fraction by CMR and by TTE	0.44	0.001

TTE: transthoracic echocardiography, CMR: cardiac magnetic resonance, 3D VCA: three-dimensional vena contracta area, DFR: diastolic flow reversal, RV: regurgitant volume, RF: regurgitant fraction, EDV: end diastolic volume, ESV: end systolic volume, LV: left ventricle, r_s_: Spearman’s correlation coefficient.

## Data Availability

The data presented in this study are available on request from the corresponding author.
